# Experimental Infection of Ferrets with *Bartonella henselae*: In Search of a Novel Animal Model for Zoonotic Bartonellosis

**DOI:** 10.3390/pathogens14050421

**Published:** 2025-04-26

**Authors:** Erin Lashnits, Cynthia Robveille, Pradeep Neupane, Toni Richardson, Keith Linder, Gabriel McKeon, Ricardo Maggi, Edward B. Breitschwerdt

**Affiliations:** 1 School of Veterinary Medicine, University of Wisconsin-Madison, 2015 Linden Dr., Madison, WI 53706, USA; 2 Intracellular Pathogens Research Laboratory, Comparative Medicine Institute, College of Veterinary Medicine, North Carolina State University, 1051 William Moore Dr., Raleigh, NC 27607, USA; cmrobvei@ncsu.edu (C.R.); pneupan@ncsu.edu (P.N.); tonigrichardson21@gmail.com (T.R.); rgmaggi@ncsu.edu (R.M.); ebbreits@ncsu.edu (E.B.B.); 3 Oxford Veterinary Hospital, Oxford, NC 27565, USA; 4 Department of Population Health and Population Biology, College of Veterinary Medicine, North Carolina State University, 1051 William Moore Dr., Raleigh, NC 27607, USA; linderpathology@gmail.com; 5 Duke Department of Pathology, Duke University School of Medicine, Durham, NC 27710, USA; gabriel.mckeon@duke.edu

**Keywords:** cat scratch disease, bartonellosis, *Bartonella henselae*, vector-borne disease, flea-borne disease, zoonotic disease, zoonosis, animal model, experimental infection, ferret, cat flea, *Ctenocephalides felis*

## Abstract

*Bartonella henselae* is an important zoonotic pathogen with a wide range of clinical manifestations in humans. Despite advances in understanding its pathogenesis, there is no broadly applicable laboratory animal model for bartonellosis. This study aimed to assess the potential utility of an experimental model of chronic *B. henselae* infection using ferrets, a species previously utilized in various human pathogen studies. Six ferrets (n = 6) were divided into three groups: a control group (n = 2), a low-dose infection group (n = 2), and a high-dose infection group (n = 2). The two infection groups were inoculated intradermally with 10^5^ (low dose) and 10^9^ (high dose) CFU/mL *B. henselae*, respectively. Clinical signs, serological responses, and bacteriological findings were monitored over seven weeks; ferrets were then euthanized and tissues were examined histologically. Only minimal or transient systemic clinical signs and laboratory abnormalities developed in *B. henselae* inoculated ferrets. The high-dose group seroconverted to *B. henselae* antigen within two weeks, maintaining elevated titers throughout the study. Histopathological examination revealed that four *B. henselae*-infected ferrets had notable microscopic inflammatory lesions in the liver parenchyma (3/4), heart (1/4), and brain (1/4); similar lesions were not observed in the tissues of the two control ferrets. Despite the presence of microscopic lesions and seroconversion in the high-dose group, bacteremia was not documented and *B. henselae* DNA was not successfully amplified by quantitative PCR from lesional organs. This pilot study demonstrated that ferrets may serve as a promising model for investigating *B. henselae* pathogenesis, diagnosis, treatment, and prevention.

## 1. Introduction

Members of the bacterial genus *Bartonella* are important emerging pathogens in humans worldwide. There are over 45 named species, more than half of which have been associated with human and animal diseases [1,2,3]. One of the most common zoonotic species is *Bartonella henselae*, which has also been shown to infect a variety of other incidental mammal hosts [2,3,4].

*B. henselae* is distributed globally, and transmitted among its reservoir host, the domestic cat, via the cat flea *Ctencephalides felis*. The prevalence of infection in cats is variable depending on the cats’ age, lifestyle, and geographic location, with ranges from <1% through >65%; highest prevalence occurs in free-roaming cats under one year old, particularly in warm and humid locations with high year-round flea burdens [5]. Transmission to humans typically occurs when infected flea feces contaminate a wound—typically a cat scratch or bite [1].

Clinical manifestations of zoonotic *B. henselae* infection in humans can range from mild to life-threatening and can present as acute or chronic [1,4,6]. *Bartonella* spp. are associated with culture-negative endocarditis in up to one-quarter of cases—making *Bartonella* spp. the second most common cause of culture-negative endocarditis—with mortality rates up to 30% [7,8,9]. Other sequelae attributed to infection with zoonotic *Bartonella* species include the typical manifestations of acute cat scratch disease (CSD) [1,10,11,12] and neuroretinitis/optic neuritis [13,14,15], but also “atypical” manifestations such as severe febrile illness [1,16,17,18,19], neurological manifestations including encephalitis/meningitis (as recently reviewed) [20], and other chronic, sometimes non-specific symptoms or pathologies that can range from mild to severe [1,21,22,23,24]. Because of this range of manifestations, including many currently under study, one or more laboratory animal models that mimic human disease would be extremely valuable.

Despite considerable advances in our understanding of the pathogenesis, immune response, and transmission of *B. henselae* through in vitro and epidemiological studies, there remains no broadly applicable laboratory animal model of bartonellosis. Animal models of human diseases provide the bedrock of much of the current understanding of cell tropism and pathogenesis and are essential for the development and licensure of drugs and vaccines. A laboratory animal model of bartonellosis could be used to understand pathogenesis, evaluate diagnostic testing methods and treatment responses, and investigate modes and routes of transmission and prevention of infection using vector control approaches or vaccine efficacy studies.

Previous studies have used cats experimentally infected with *B. henselae* and other *Bartonella* spp. to draw conclusions regarding human disease and transmission [25,26,27,28,29,30,31,32,33,34,35,36,37,38,39]. However, the use of cats as an experimental model for *B. henselae* in humans is problematic. As a primary reservoir host for *B. henselae*, cats have different evolutionarily determined biological responses to infection compared to humans, so their clinical signs and immunological responses would not be expected to accurately recapitulate human disease. In addition, optimal diagnostic testing strategies, as well as maintenance and transmission of infection, likely differ between the cat reservoir host and incidentally infected humans. These factors limit the utility of the cat as an experimental model for studying disease immunopathogenesis, as well as transmission pathways and vaccination development for the prevention of *B. henselae* infection in people.

Other animal models of clinical bartonellosis have been elusive. Mice have been used to study the cellular and immunological basis of infection, but investigators have often infected them with mouse-adapted *Bartonella* spp. rather than *B. henselae* [40,41,42,43]. Mouse models of *B. henselae* have been attempted to study immunological and pathological responses to infection [42,43,44,45], but have proved not to be broadly useful in modeling clinical disease or transmission due to the difficulty of inducing infection and lack of clinical manifestations in immunocompetent mice [42,46,47,48]. Zebrafish embryos have been successfully used to identify virulence factors and define molecular mechanisms of *B. henselae* pathogenesis, but are clearly not useful in recapitulating human disease or assessing transmission [49]. As incidental hosts with disease manifestations in natural infections similar to humans, naturally infected dogs are considered a promising animal model of human disease [50,51,52,53,54,55]. Dogs experimentally infected with other species of *Bartonella* have also been proposed as animal models for human disease, but the laboratory use of dogs—particularly in terminal studies—is undesirable due to ethical considerations for this companion animal species [56,57].

The ferret has been used in numerous experimental models to study human pathogens, including most notably influenza, but also bovine tuberculosis, measles, chronic wasting disease, and, most recently, SARS-CoV-2 [58,59,60,61,62,63,64,65,66,67]. As a commonly utilized laboratory animal species, if ferrets are susceptible to *B. henselae* infection they would be an attractive surrogate species with which to study various aspects of *B. henselae* transmission and pathogenesis and to assess treatment efficacy. Exposure to various *Bartonella* spp. has been found in a variety of mustelids (the family of carnivores into which ferrets fall), including otters [68,69,70], martens [71], minks [72], and badgers [73,74], and *B. henselae* specifically was reported in sea otters [69]. While there is a single case report proposing a pet ferret as the source for *B. henselae* neuroretinitis, ferrets have not otherwise been investigated as hosts of either *B. henselae* or other *Bartonella* spp. that induce zoonotic infections [75].

The overall objective of this study was to investigate a novel experimental model of *B. henselae* bartonellosis using the ferret. The development of such an animal model could ultimately lead to improvements in the diagnosis, treatment, and prevention of bartonelloses in animals and human patients. The specific aims of this pilot study were to describe clinical signs, histopathologic changes, longitudinal serological responses, and bacteriological findings after a single intradermal inoculation of *B. henselae* in healthy ferrets.

## 2. Materials and Methods

### 2.1. Experimental Animals

One male neutered and one female spayed ferret, aged 16–19 weeks, were included in each group (n = 6). Ferrets were obtained from a commercial source (Triple F Farms Inc., Gillett, PA, USA) and were neutered and descented prior to arrival.

### 2.2. Housing and Husbandry

**Figure 1:**
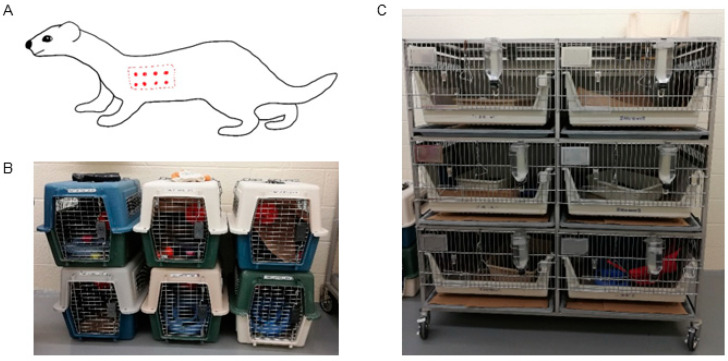
Animals and environment. (**A**) Diagram of inoculation sites. Dotted line indicates shaved area (bilateral), red points indicate injection sites (0.5 mL per side). (**B**) Individual carriers for use during cage cleaning and handling. (**C**) Arrangement of individual cages in animal facility.

Ferrets were housed in individual cages (Figure 1C) with a 12 h day–night cycle in a temperature- (70–74 °F) and humidity- (30–70%) controlled room. Ferrets were allowed free access to water and a maintenance diet (Marshall BioResources, North Rose, NY, USA). All cages contained a litter pan and toys for environmental enrichment. When removed from their cages for cage cleaning, each ferret was placed into an individual portable carrier assigned to that ferret (Figure 1B) to minimize the potential for carryover of bacteria or bacterial DNA between ferrets during handing. Because of ferret temperament and to ensure the safety of facility staff, all ferret handling was carried out with bite-proof gloves.

### 2.3. Ethical Statement

This study was carried out in strict accordance with the recommendations in the Guide for the Care and Use of Laboratory Animals of the National Institutes of Health. The study was approved by the North Carolina State University Institutional Animal Care and Use Committee (IACUC #18-175-B). All blood draws were performed under inhaled isoflurane anesthesia (isoflurane 1–2% to effect via mask), and all efforts were made to minimize suffering.

### 2.4. Study Design

For this pilot study, there were three groups of two ferrets each (one male and one female): a control group, inoculated with 1 mL sterile saline intradermally (ID); a low-dose group, inoculated with 10^5^ CFU/mL *B. henselae* ID; and a high-dose group, inoculated with 10^9^ CFU/mL *B. henselae* ID. The intradermal route and inoculation doses were chosen based on previous experiments with cats and dogs [26,33,76]. Ferrets were grouped by sex and randomly allocated into groups. Researchers performing *B. henselae* testing were blinded to the group status for all samples.

**Figure 2:**
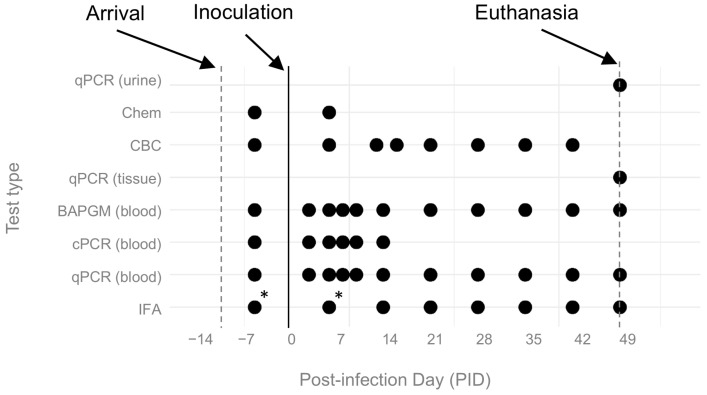
Timeline of experimental events. Test type is shown on the y-axis and study day on the x-axis. Dots indicate date on which the given test occurred. * indicates missing tests due to a lack of serum. Complete blood counts (CBCs) scheduled for PID 14 were performed on PID 13 or 16 due to animal handling and sampling constraints. Chem = serum chemistry panel; CBC = complete blood count; qPCR = quantitative PCR; BAPGM = *Bartonella* alpha proteobacteria growth medium enrichment culture with qPCR; cPCR = conventional PCR; IFA = *B. henselae* immunofluorescent antibody assay.

Details of the study timeline are provided in Figure 2. Briefly, ferrets were purchased from a commercial provider and arrived at the NC State University Laboratory Animal Resources (NCSU LAR) facility 12 days prior to inoculation (PID -12). Following 4 days of acclimation in which the ferrets were not handled, physical exams, ear cytology, and routine bloodwork (CBC and serum chemistry) were performed and prophylactic ectoparasite preventative (15 mg selemectin topical, Revolution® topical solution for puppies and kittens, Zoetis, Parsipanny, NJ, USA) was administered topically. Ferrets’ clinical health status was monitored throughout the experiment by facility staff and/or investigators. All ferrets were inoculated on a single day (PID 0), and serial blood samples were collected over the following 7 weeks. Ferrets were sedated with midazolam and butorphanol for the initial physical exam; for each subsequent sample collection, they were sedated with inhaled isoflurane to effect. Temperatures were taken at the beginning of sedation via a rectal thermometer. Ferrets were euthanized on day 49 following infection (PID 49).

### 2.5. Inoculum and Animal Procedures

For the *B. henselae* inoculum, *B. henselae* San Antonio 2, isolated from a naturally infected cat (NCSU strain 95 FO-099), was plated on trypticase soy agar plates supplemented with 5% defibrinated rabbit blood (BBL, Cockeysville, MD, USA) and incubated for 7 days at 35 °C in the presence of 5% CO_2_ and 99% relative humidity [77]. Colonies were then suspended in 1 mL sterile saline. Bacterial concentration was confirmed with qPCR. Suspension of colonies into sterile saline and qPCR were performed on the day of inoculation of ferrets (PID 0). After inoculation of the ferrets, the remaining inocula were stored at −80 °C.

For infection, each ferret was moved individually from its cage to a box, sedated via inhaled isoflurane to effect, and then moved to the hood where sedation was maintained with isoflurane via a mask. A small section of fur was shaved on both sides of the thorax for the inoculation sites (Figure 1A). Each inoculum was divided into 0.5 mL aliquots and 2 aliquots were injected intradermally in multiple locations within the shaved site, on both sides, for a total of 1 mL inoculum per ferret (Figure 1A). Ferrets were monitored until they were awake and then returned to individual cages.

For blood sample collection, ferrets were sedated with inhaled isoflurane to effect. Blood was collected via the cranial vena cava. After collection, blood was transferred into an ethylenediaminetetraacetic acid (EDTA)-treated tube (Greiner Bio-One VACUETTE™ K2EDTA Blood Collection Tubes; Kremsmünster, Austria) and an untreated tube to allow the blood to clot prior to serum separation. At the endpoint of the experiment, ferrets were sedated with isoflurane, urine was collected via cystocentesis, and ferrets were euthanized by intracardiac injection with an overdose of pentobarbital.

Autopsies were performed immediately following euthanasia by a board-certified veterinary pathologist (K.L.). New sterile scalpel blades were used for each ferret and were sterilized with heat in between tissues to minimize the potential for DNA carryover between animals and between tissues within each animal. The following tissues were sampled, with one sample stored frozen and one fixed in 10% neutral buffered formalin for histology analysis: bone, bone marrow, brain, diaphragm, ear tip, eyeball, heart, kidney, liver, mandibular and mesenteric lymph nodes, skin (inoculation site, intrascapular and caudal dorsum), striated skeletal muscle, spleen, thymus, adrenal gland (except for the male ferret in the high-dose group), lung (except for the female ferret in the low-dose group), and thyroid gland (except for both control ferrets). The following additional tissues were available for all except the male ferret in the low-dose group: gall bladder, pancreas, salivary gland, stomach, small intestine, and caecum/colon. Fixed tissues were processed, embedded in paraffin, sectioned at 5 µm thickness, and stained with hematoxylin and eosin. Selected tissues were also stained with Gram and Warthin–Starry. Histology evaluation was performed by a board-certified veterinary pathologist (C.R.) in a blinded manner.

For all animal procedures, the order in which ferrets were removed from their cages for procedures was standardized: the control group first, followed by the low-dose group, then the high-dose group. This was carried out to minimize the likelihood of the spread of bacteria or bacterial DNA from infected to uninfected ferrets. An exception occurred during inoculation when one low-dose group ferret was removed from the cage for experimental procedures prior to the second control group ferret (female) due to constraints on LAR staff and animals being amenable to handling.

### 2.6. Humane Endpoints

Specific criteria were developed to limit suffering in experimental animals. The experiment was planned for a duration of 7 weeks, at which time all animals would be humanely euthanized as described above to determine chronic infection status and for evidence of chronic infection in *B. henselae* inoculated ferrets. There were 6 ferrets used, and all 6 were euthanized as planned after 7 weeks; none were found dead.

All ferrets were monitored closely for an initial 2 weeks following infection (or sham infection). Animals had physical examinations including rectal temperature, observation of attitude, appetite changes, presence of nausea, hydration status, examination of inoculation site, and evaluation for skin lesions. These exams were performed and recorded twice daily by a veterinary technician, with results reviewed by study personnel (E.L.) daily. Planned blood draws for infection and disease monitoring followed the pre-determined schedule, with increased frequency planned for any ferrets who developed more severe clinical signs. No animals required additional blood draws or any supportive care during the experiment. With no signs of disease at the 2-week timepoint, monitoring continued for another approximately 5 weeks with the above-outlined technician evaluations every 2–3 days, and weekly blood draws for CBC and *Bartonella* testing.

Supportive care planned prior to study initiation included the following: Ferrets showing a reduced appetite would be enticed to eat with a high-calorie diet supplement (Carnivore Care by Oxbow Animal Health, Omaha, NE, USA). Ferrets that showed evidence of dehydration on physical examination would be treated supportively with subcutaneous fluids. If ferrets developed signs of severe disease, euthanasia with a complete post-mortem exam would be performed to determine the cause of illness and specific pathology. Criteria for severe disease included, but were not limited to, the following parameters: fever in excess of 103.5 °F persisting longer than 72 h; refusal of food for 72 h; and progressive dehydration over 72 h (based on the clinical assessment of the veterinary staff, including skin turgor, tear film, and mucous membrane appearance).

### 2.7. Diagnostic Procedures

According to the schedule in Figure 2, CBCs and chemistry panels were performed at the Clinical Pathology Laboratory at the Veterinary Hospital at North Carolina State University. Physiologic reference intervals for ferret CBC and chemistry values were retrieved from the International Species Information System [78]. The remaining blood samples were then stored and refrigerated. *Bartonella* testing was performed in two batches (mid-point and endpoint) to minimize variation.

For *Bartonella* PCR, DNA was extracted from EDTA anti-coagulated blood and fresh frozen tissue samples using a Qiagen DNeasy^®^ Blood and Tissue kit (Qiagen, Valencia, CA, USA) following the manufacturer’s protocols. DNA yield and quality were assessed by spectrophotometry (Nanodrop, Wilmington, DE, USA). For tissue samples obtained during autopsy (cerebrum, heart, liver, spleen), two approximately 25 mg samples from each organ were tested by qPCR. qPCR was performed using 5 μL of each DNA sample.

*Bartonella* genus-specific conventional PCR was performed on ferret blood DNA extracts using primers designed to amplify the sequence in the *Bartonella* 16S–23S internal transcribed spacer (ITS) region as previously described [79]. For conventional PCR, primers 325s (CCTCAGATGATGATCCCAAGCCTTCTGGCG) and 1100as (GAACCGACGACCCCCTGCTTGCAAAGCA) were used.

Ferret blood and tissue DNA extracts were screened for the presence of *Bartonella* spp. DNA using qPCR, with primers targeting the 16S–23S intergenic transcribed spacer (ITS) region of *Bartonella* species as described previously [80], in conjunction with a BsppITS500 FAM-labeled hydrolysis probe (TaqMan, Applied Biosystems, Foster City, CA, USA). Amplification was performed in a 25 µL final volume reaction containing 12.5 µL of MyTaq Premix (Bioline, Memphis, TN, USA), 0.2 µL of 100 µM of each forward primer and reverse primer (IDT^®^ DNA Technology, Coralville, IA, USA), 7.1 µL of molecular-grade water, and 5 µL of DNA from each sample tested.

Negative and positive controls for PCR were prepared using 5 µL of DNA from the blood of a healthy dog previously characterized positive dog (clinical case), respectively. Validation of positive results was performed by Sanger sequencing of amplicons followed by chromatogram evaluation and sequence alignment using Contig-Express and Align X software (Vector NTI Suite 10.1, Invitrogen Corp, Carlsbad, CA, USA). For bacterial species identification, DNA sequences were analyzed for nucleotide sequence homology at the NCBI nucleotide database using BLAST version 2.0.

Stringent processing methods were used to avoid DNA carryover during tissue processing [81]. Specifically, tissue samples were processed independently using manual DNA extraction. For all batches of DNA extractions, between 2 and 4 blank samples (water) were used as negative controls. All negative controls for DNA extractions rendered negative results on all PCR assays. DNA carryover after PCR amplification was avoided by processing each sample in three separate laboratory rooms (one for sample sorting and DNA extraction, a second for PCR processing, and a third for PCR analysis post amplification), and strict use of personal protective equipment for sample handling by laboratory personnel. All tissues were tested by qPCR.

*Bartonella* spp. bacteremia was assessed using *Bartonella* alpha proteobacteria growth medium enrichment culture with qPCR (BAPGM-ePCR), as previously described with minor modifications [82]. Briefly, for each sample, first 0.5 mL of blood-EDTA was cultured in 5 mL of BAPGM media, then incubated in 5% CO_2_ at 35 °C with 100% humidity for 21 days. Aliquots of each culture, consisting of 200 µL of blood culture, were subsampled after 7, 14, and 21 days of growth and tested by qPCR for *Bartonella* DNA, as described above. A blood sample was considered BAPGM-ePCR positive if any one or more of these 3 subsamples was qPCR positive.

For immunofluorescent antibody assay (IFA) testing, *Bartonella* antibodies were determined using cell culture-grown *B. henselae* as antigens and following standard techniques [83]. Briefly, bacterial colony isolates were passed from agar plate-grown cultures into permissive cell lines. For each antigen, heavily infected cell cultures were spotted onto 30-well Teflon-coated slides (Thermo Fisher Scientific, Carlsbad, CA, USA), air-dried, acetone-fixed, and stored frozen. Fluorescein conjugated goat anti-ferret IgG (KPL, SeraCare, Milford, MA, USA) was used to detect bacteria within cells using a fluorescent microscope (Carl Zeiss Microscopy, LLC, Thornwood, NY, USA). Serum samples were diluted in phosphate-buffered saline (PBS) solution containing normal goat serum, Tween-20, and powdered nonfat dry milk to block nonspecific antigen binding sites. Sera were first screened at dilutions of 1:16 to 1:64. All sera that were reactive at 1:64 were further tested with two-fold dilutions to 1:8192. A cutoff IgG titer of 1:64 was used to define a seroreactive IFA titer.

### 2.8. Experimental Outcomes

Two primary outcomes indicating successful infection were analyzed: first, acute *B. henselae* infection, as determined by assessment of conventional and qPCR and BAPGM ePCR on blood samples, and *B. henselae* IFA seroreactivity during the initial 4 weeks of the study. Second, chronic *B. henselae* carriage was determined by qPCR on tissue samples at the endpoint of the study (7 weeks). In addition, a secondary outcome of clinical disease was assessed by longitudinal physical examinations, along with CBC and serum chemistry panel results and histopathological changes on tissue samples at the endpoint of the study (7 weeks).

### 2.9. Statistical Methods

For this pilot study using 6 animals, descriptive results for each animal are presented. Specifically, the clinical data collected on each ferret included body temperature and body weight (shown as line graphs). Mean body temperature and body weight for all ferrets at each timepoint was calculated. Relevant parameters from complete blood counts included hematocrit, platelet count, absolute white blood cell count, absolute neutrophil count, absolute lymphocyte count, absolute eosinophil count, and absolute monocyte count. Values are shown for each ferret at each timepoint as line graphs; the mean value for each parameter at each timepoint was also calculated. Finally, relevant parameters from serum chemistry panels included albumin, globulin, glucose, blood urea nitrogen, creatinine, cholesterol, creatine kinase, alkaline phosphatase, gamma-glutamyl transferase, alanine aminotransferase, and aspartate aminotransferase. Values are shown for each ferret at each timepoint as line graphs; the mean value for each parameter at each timepoint was also calculated.

## 3. Results

### 3.1. B. henselae Infection

**Figure 3:**
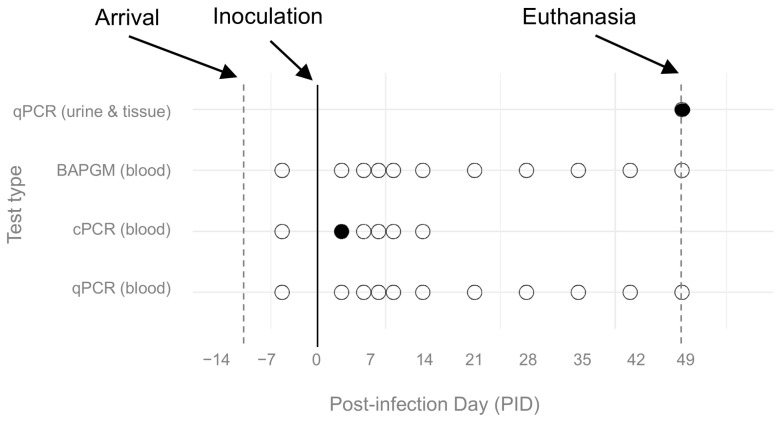
Molecular and microbiological *B. henselae* test results. Test type is shown on the y-axis and study day on the x-axis. Dots indicate the date on which the given test occurred; open dots indicate a negative test, closed black dots indicate a positive test. Positive tests included: on PID 3 whole blood from one high-dose infected ferret (male); and at the time of euthanasia on PID 49, urine from one high-dose infected ferret (female), one of two heart DNA extractions from one low-dose infected ferret (female), and one of two spleen DNA extractions from one control ferret (female). qPCR = quantitative PCR; BAPGM = *Bartonella* alpha proteobacteria growth medium enrichment culture with qPCR; cPCR = conventional PCR.

Molecular and microbiological test results are shown in Figure 3. Based on conventional and qPCR, no ferrets had *B. henselae* DNA in their blood prior to experimental infection (PID 0). Based on conventional PCR, one ferret (male) in the high-dose group had *B. henselae* DNA amplified from whole blood on post-inoculation day (PID) 3. Amplicon sequence from this ferret shared 100% homology (132/bp) with *B. henselae* SA2 (GenBank accession # AF369529), the species and strain type inoculated. Using qPCR on DNA extracted from whole blood, there were no other positive samples from any ferret at any timepoint. Based on BAPGM enrichment blood culture, viable *B. henselae* was not isolated, and all BAPGM-ePCR blood culture assays were negative, from blood samples from all ferrets at all timepoints both prior to and following experimental infection.

Samples were obtained at autopsy on PID 49 (Figure 3). *B. henselae* DNA (70/70 bp identical with *B. henselae* strain SA-2) was amplified and sequenced from two of eight tissues: one of two heart DNA extractions from a female low-dose infected ferret, and one of two spleen DNA extractions from a female control ferret. The remaining tissues, including two DNA extractions each from the heart, cerebrum, liver, and spleen, were *Bartonella* spp. qPCR negative. DNA extractions from urine samples obtained via cystocentesis at the time of euthanasia were all *Bartonella* spp. qPCR negative, except for the sample from the female high-dose ferret.

**Figure 4:**
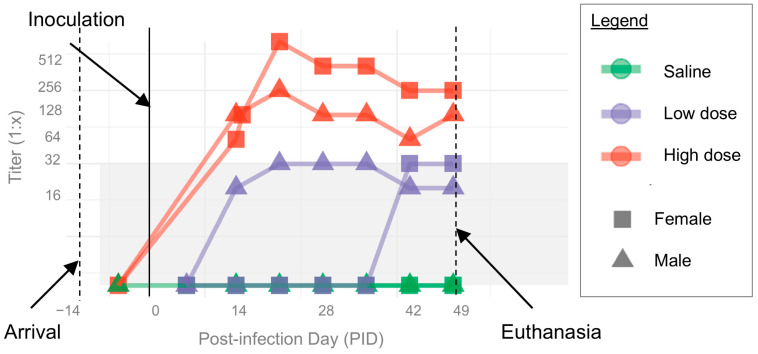
*B. henselae* immunofluorescent antibody (IFA) seroreactivity. Colored lines and points indicate individual ferrets; colors show groups (green = saline, purple = low dose, red = high dose) and points show sex (squares = female, triangles = male). Gray shading indicates titers considered below the predetermined two-dilution cutoff point for positive seroreactivity (1:64). Titers < 1:16 were set at 1 for visualization.

IFA titers for all ferrets are shown in Figure 4. Based on IFA, all ferrets were *B. henselae* seronegative (IFA titer < 1:16) prior to experimental infection (PID 0). Following inoculation, both ferrets in the high-dose group seroconverted to *B. henselae* antigen by PID 14. Titers remained elevated through the endpoint of the study, with peak titers of 1:1024 and 1:256 for the female and male high-dose group ferrets, respectively. Low-dose group ferrets had consistently increased IFA titers (1:32) that did not reach the predefined cutoff point to be considered seroreactive. However, both control ferrets had no change in their titers from baseline (<1:16).

### 3.2. Histopathologic Lesions

**Figure 5:**
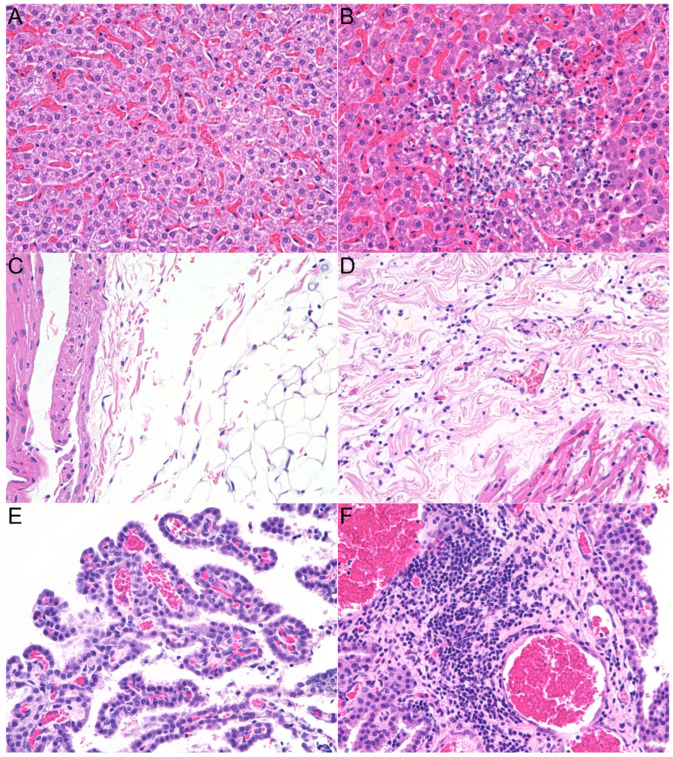
Microscopic findings at ×20 objective, hematoxylin, and eosin. Liver parenchyma (**A**), epicardium (**C**), and choroid plexus (**E**) are unremarkable in the control ferret. Mixed-cell inflammatory infiltrate associated with loss of hepatocytes in the high-dose male ferret (**B**). Lymphoplasmacytic epicarditis in the high-dose female ferret (**D**). Lymphoplasmacytic plexus choroiditis in the high-dose male ferret (**F**).

The four *B. henselae* infected ferrets had notable microscopic inflammatory lesions in the liver parenchyma (3/4), heart (1/4), and brain (1/4); similar lesions were not observed in the tissues of the two control ferrets (Figure 5A,C,E). One low-dose ferret and both high-dose ferrets had a few small (≤200 µm) foci, randomly distributed among the hepatocytes, composed of a variable number of neutrophils, lymphocytes, plasma cells, and histiocytes (Figure 5B). In addition to the liver lesions, the high-dose female ferret had mild multifocal lymphoplasmacytic myocarditis and epicarditis (Figure 5D), and the high-dose male ferret had mild lymphoplasmacytic plexus choroiditis (Figure 5F). Given the positive qPCR result in the heart of the low-dose female ferret, which was initially unremarkable at the microscopic evaluation, three additional deep sections (100 µm between sections) were examined. No evidence of lesions, including inflammation, was observed in these additional sections. Splenic extramedullary hematopoiesis was minimal in both control ferrets, mild in three infected ferrets, and moderate in one high-dose ferret (male). One low-dose ferret (male) had a single eosinophilic granuloma (250 µm × 200 µm) with a Splendore–Hoeppli reaction in one lymph node; this finding was likely incidental. No pathogens were identified with Gram or Warthin–Starry staining in lesional tissues. There were no significant lesions or differences between control ferrets versus infected ferrets in the other evaluated tissues. Distinct from the hepatic parenchymal lesions in the three infected ferrets, there were also lymphocytes and sometimes plasma cells observed in the portal tract of all four infected ferrets, and both control ferrets. There was no correlation between the severity of the portal inflammation and the status of the ferrets.

### 3.3. Clinical Disease

**Figure 6:**
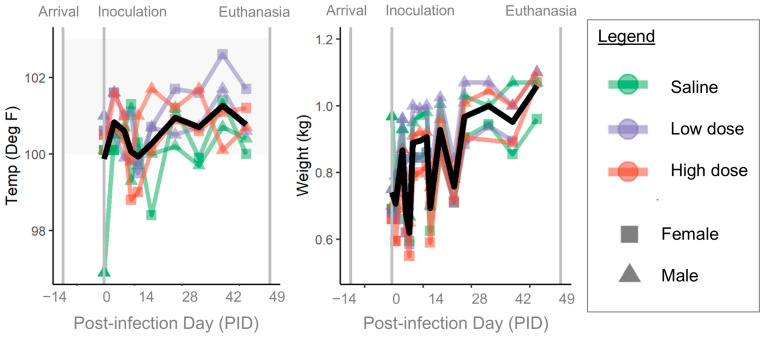
Physical exam findings. Colored lines and points indicate individual ferrets; colors show groups (green = saline, purple = low dose, red = high dose) and points show sex (squares = female, triangles = male). Gray shading indicates reference ranges. On each plot, left-most gray vertical line shows the day of arrival, middle gray vertical line shows the day of inoculation, and right-most gray vertical line shows the day of euthanasia. Black line shows mean value for each measurement. Temp = body temperature.

Body temperature and weight throughout the experiment are shown in Figure 6. All ferrets had normal physical exams including normal vital signs prior to experimental infection (PID -8, -5, -1) and prior to inoculation on PID 0. Ferret weights fluctuated but generally increased over the course of the study. No ferret developed a fever; however, one ferret (low-dose female) had a temperature of 102.6 °F on day 35 after inoculation [reference range of 100−104 °F].

The two high-dose inoculated ferrets developed raised erythematous lesions at the injection sites the day after inoculation (PID 1). These lesions improved quickly without treatment, initially resolving by PID 3−4. However, skin lesions reappeared by PID 9−14, persisting through PID 18−20 before resolving without treatment. Initially, the injection site lesions were mildly raised and erythematous, and some sites formed small scabs. Later, the skin lesions were erythematous but flat, and mildly pruritic. Other than the skin lesions, all but one ferret continued to have normal physical exams throughout the duration of the study. One ferret (high-dose female) was noted to cough on PID 8 and had mild serous ocular discharge bilaterally on Day 9. On PID 16, this ferret was shaking, and on PID 17−18, noted to be quieter and less active than normal in the cage.

**Figure 7:**
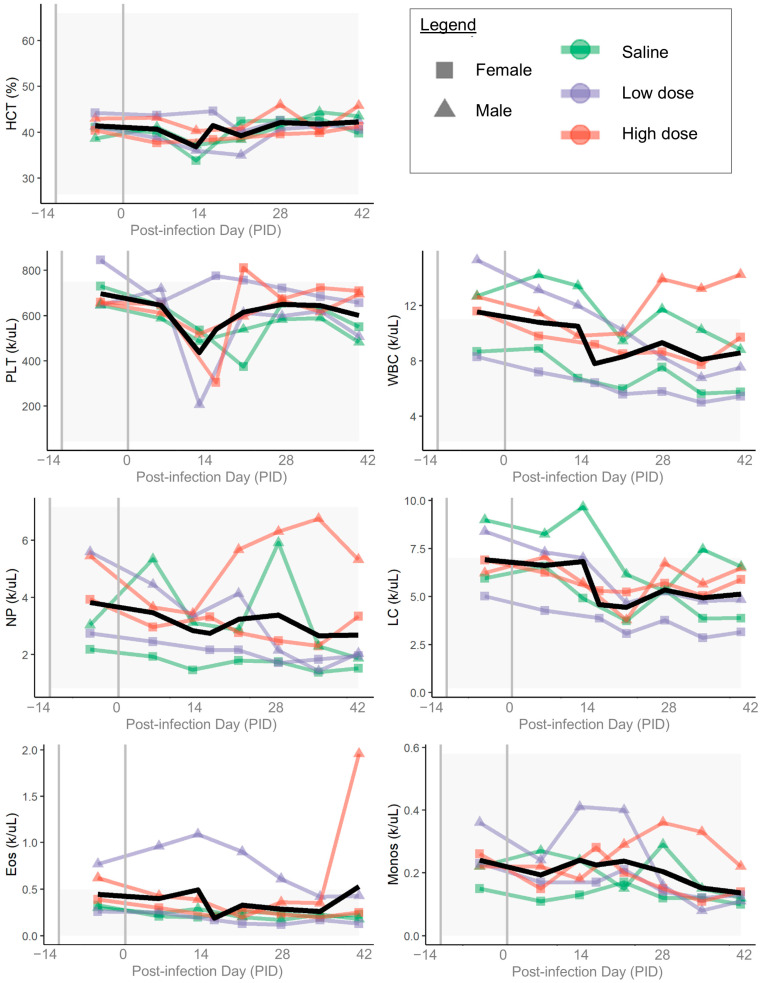
Complete blood count (CBC) results. Colored lines and points indicate individual ferrets; colors show groups (green = saline, purple = low dose, red = high dose) and points show sex (squares = female, triangles = male). Gray shading indicates reference ranges. On each plot, left-most gray vertical line shows the day of arrival, and right-most gray vertical line shows the day of inoculation. Black line shows mean value for each measurement. HCT = hematocrit; PLT = platelet count; WBC = absolute white blood cell count; NP = absolute neutrophil count; LC = absolute lymphocyte count; Eos = absolute eosinophil count; Monos = absolute monocyte count.

Relevant complete blood count parameters from each ferret are shown in Figure 7. There were no clinically relevant patterns of change in hematocrit or erythron indices throughout the 49-day course of the study. White blood cell counts and platelet counts had a general decreasing trend through the course of the study for most ferrets (control and infected), though there was individual variability. There was persistent mild eosinophilia in one low-dose male ferret; this ferret also had a single eosinophilic granuloma in one lymph node collected at autopsy on PID 49.

**Figure 8:**
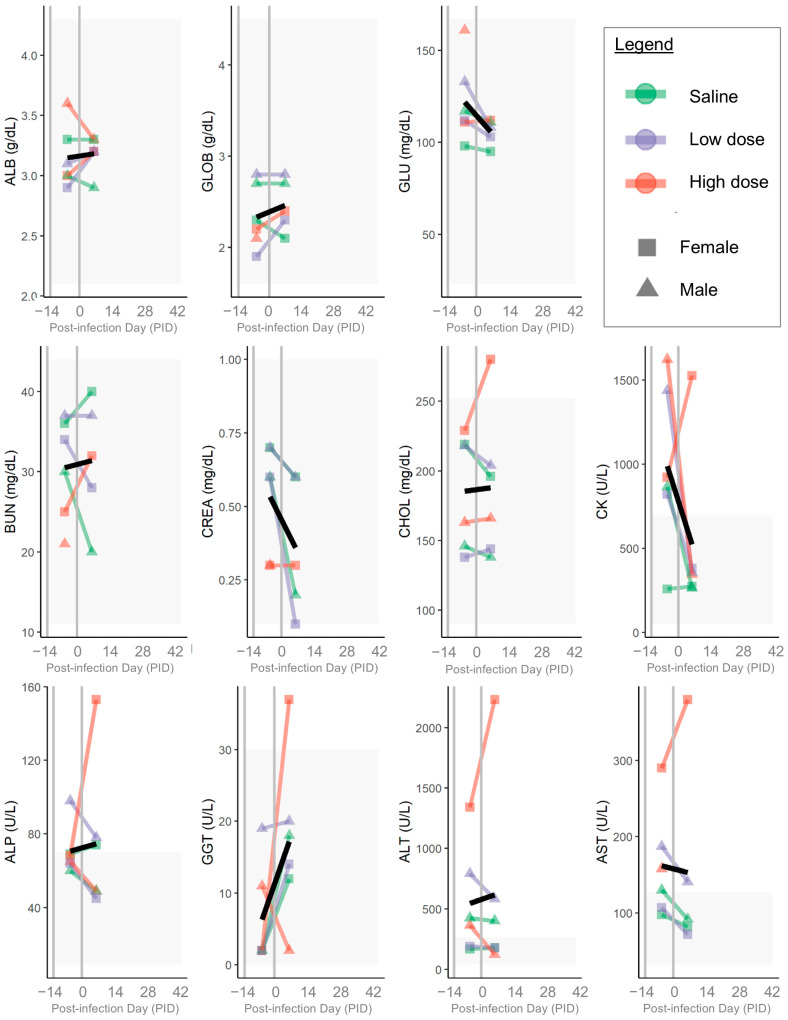
Serum chemistry panel results. Colored lines and points indicate individual ferrets; colors show groups (green = saline, purple = low dose, red = high dose) and points show sex (squares = female, triangles = male). Gray shading indicates reference ranges. On each plot, left-most gray vertical line shows the day of arrival, and right-most gray vertical line shows the day of inoculation. Black line shows mean value for each measurement. ALB = albumin; GLOB = globulins; GLU = glucose; BUN = blood urea nitrogen; CREA = creatinine; CHOL = cholesterol; CK = creatine kinase; ALP = alkaline phosphatase; GGT = gamma-glutamyl transferase; ALT = alanine aminotransferase; AST = aspartate aminotransferase.

Relevant serum chemistry parameters from each ferret are shown in Figure 8. Serum chemistry abnormalities prior to inoculation included elevations of ALP, ALT, and/or AST for multiple ferrets, including one (high-dose female) with a severe elevation of ALT; the ALT, AST, ALP, and GGT in this ferret all increased following inoculation. The CK was elevated prior to inoculation in most (5 of 6) ferrets. Otherwise, there were no clinically relevant abnormalities on serum chemistry panels (Figure 8) or electrolytes either before or after inoculation.

## 4. Discussion

In this pilot study, ferrets became acutely infected with *B. henselae* following a single 1 mL intradermal inoculation of 10^9^ CFU/mL, seroconverting within 2 weeks after inoculation, and maintaining positive titers throughout the study period of 7 weeks after inoculation. *B. henselae* DNA was detected in blood 3 days after inoculation in one ferret and in the heart tissue of another ferret 7 weeks after inoculation; however, *B. henselae* DNA was not detected in blood or organs showing histopathological lesions on post-mortem examination of infected ferrets 7 weeks after inoculation. This extremely rare detection of *B. henselae* DNA from inoculated, seroreactive ferrets may reflect complete clearance of the pathogen via a rapid and effective immune response in conjunction with the development of *B. henselae*-specific circulating antibodies. Alternatively, it is also possible that the immune response to infection decreased bacteremia to below the level of qPCR detection, or induced changes in the bacteria to cause biofilm formation [84], but did not actually clear the infection. If that is the case, a longer follow-up period, or comorbid illness/immunosuppression, may have allowed us to document recrudescent or chronic infection by increases in the level of bacteremia above the level of detection [85,86].

The IgG antibody titers were higher in the high-dose group (up to 1:1024) compared to the low-dose group (1:32), indicating a possible dose-dependent antibody response following the inoculation. To demonstrate the specificity of this immune response, there was no change from baseline (<1:16) in the control ferrets. While IgG titer dynamics and [87,88] variability among individual cats following experimental *B. henselae* infection have been previously reported [28,76], at least one previous study reported a lack of correlation between inoculum dose and peak-IgG titer [26]. Whether the apparent dose-dependent humoral immune response shown here in ferrets occurs during natural *B. henselae* infection of incidental hosts with *B. henselae* is unknown, though high-IgG titers are expected in human *Bartonella* spp. endocarditis cases [7].

Despite minimal to no systemic or clinical signs of illness, both high dose and one of the two low doses inoculated infected ferrets had microscopic lesions that were not found in the control ferrets at the end of the study. The liver was the most commonly affected organ. Three out of four infected ferrets had hepatic lesions, characterized by small (≤200 µm) mixed-cell inflammatory foci in the parenchyma. Hepatitis and micro-abscesses in the liver parenchyma have been reported in cats infected with *B. henselae* by the intravenous (IV) route [26,38] and in a naturally infected dog [52]; it is also a well-known atypical manifestation of *Bartonella henselae* infection in children [87,88] and has been reported in both immunocompromised and immunocompetent adult humans [89,90,91,92,93,94,95,96,97].

In addition to the liver lesions, one high-dose ferret (female) had mild lymphoplasmacytic infiltrates in the heart and epicardium, whereas one low-dose ferret had *B. henselae* DNA PCR amplified and sequenced from cardiac tissue, despite no discernible histopathological changes. The heart, especially the aortic valve, is a common location for *B. henselae* localization in people and dogs with culture-negative endocarditis [7]. No evidence of endocarditis was seen in the infected (or control) ferrets. It is possible that pre-existing valve defects increase the risk for endocarditis after *Bartonella* spp. infection, as reported in some human and dog cases [7]. It is also possible that the infected ferret had myocardial inflammation despite clearance of the pathogen. Myocarditis has been reported in feline experimental infections by IV route [26,38], as well as in natural infection in cats [98], Florida pumas [99], and one dog [100]. *Bartonella* infection can be associated with neutrophils, lymphocytes, plasma cells, and macrophages. Lymphoplasmacytic inflammation, such as described here, can be the primary change or a chronic change secondary to a previous suppurative inflammation. Without cardiac tissue samples from earlier timepoints, it is unknown whether there was an initial neutrophilic component to the inflammation seen. In one experimental study of *B. henselae*- and *B. clarridgeiae*-infected cats, the myocardial inflammation found in over half of infected cats (8 or 13) at over one year after initial infection was reported as a mixture of lymphocytes and plasma cells, supporting this as a chronic sequela of *Bartonella* spp. infection [38]. While lymphoplasmacytic inflammation is not specific to *Bartonella* spp. infection or even infectious causes, it was not seen in the heart samples of control ferrets.

Lymphoplasmacytic infiltrates were visible in the choroid plexus in one high-dose ferret (male). This histology finding has been described in a dog infected with *B. henselae* SA2 strain by subcutaneous inoculation [57]. In experimentally infected cats, neurological signs such as nystagmus and/or seizures have been reported, and *Bartonella* spp. DNA has been amplified in the brain from both symptomatic and asymptomatic cats [25,38]. In the past few years, *B. henselae* has been implicated in an increasing number of humans with neurological, cognitive, and/or neuropsychiatric symptoms [20]. Meningitis and/or encephalitis have been reported in humans, including rarely with a fatal outcome [20,101,102]. Diagnostically, cerebrospinal fluid can be normal or contain elevated proteins in patients with *B. henselae* neurobartonellosis [103,104].

The ferrets receiving high-dose inoculation in this study did develop local skin reactions, though no histopathological changes persisted in the inoculated skin 7 weeks after infection. Local transient skin lesions have been previously reported in experimentally infected cats [25,36,37] and dogs [56] and are known to occur in human infection [105].

For reasons that remain unknown, pathological lesion severity frequently does not correlate with microbiological isolation, PCR amplification, or organism visualization in both natural and experimental *B. henselae* infections involving immunocompetent individuals. For this reason, deriving definitive conclusions on the histopathological changes induced in ferrets by *B. henselae* infection in this study cannot be made because the various histopathological lesions were visible in only one or a few infected ferret(s). More studies are needed to understand the variability in histological lesions, the duration and pathogenicity associated with prolonged infections, and the ferrets’ potential to mimic natural disease in animals and human patients.

Unexpectedly, *B. henselae* SA2 DNA was amplified from the spleen of one saline-inoculated control ferret. Although DNA carryover or contamination are possibilities, all DNA extraction and qPCR negative controls remained negative throughout the study. *Bartonella* infection occurring at the commercial provider, prior to procurement of the ferrets and housing in the research facility, is also possible, though all ferrets were PCR and BAPGM negative, as well as seronegative, prior to experimental inoculation. If occult infection prior to procurement was the cause of the B. henselae DNA in the control group ferret spleen, this infection does not appear to have induced seroreactivity via IFA.

Another possible explanation for the positive qPCR from the control group ferret includes horizontal direct transmission: the potential for horizontal direct transmission of *Bartonella* spp. among dogs in a vector-free laboratory animal setting was recently reported after experimental infection with *Rickettsia rickettsii* [106]. Recently, occult infection and reactivation have been further supported in a recent study indicating that mice maintained in a laboratory animal facility for many generations appeared to be infected with *B. henselae* [107], and other reports suggesting reactivation of latent *B. henselae* infection following COVID in people [108,109]. Despite being a vector-borne intravascular and endotheliotropic bacteria, the *B. henselae* SA2 strain used in this study has also been shown to remain viable in air-dried blood, milk, saline, serum, and urine following 7 days of desiccation [110]. One urine sample from one high-dose ferret at the time of euthanasia (PID 49) was also qPCR positive, indicating that *Bartonella* spp. DNA was present in the urine of at least one of the inoculated ferrets. Although ferrets were housed individually throughout the experiment, feces, and urine were not maintained solely within the individual cages. Also, ferrets were routinely handled in a common location during cage cleaning, examinations, and blood draws. Therefore, transmission through aerosolization, direct contact with feces and urine, or inadvertent inoculation of bacteria from contamination of environmental surfaces during blood draw could have resulted in infection of the control ferret. This could have occurred late in the experiment and, prior to documentation of seroconversion, earlier with a minimal immune response due to extremely low-dose inoculation or as in the case of young cats inoculated with *B. henselae* orally, seroconversion did not occur [36]. This control ferret did not have any of the abnormal histopathological lesions seen in the infected ferrets, making symptomatic or clinically relevant infection less likely. Based upon these observations, if this experimental model is pursued in the future, uninfected controls should be physically separated and maintained in a manner to avoid exposure to infected fomites in experimental colonies, particularly when procedures that could inoculate microscopic infectious material are planned (e.g., blood draws).

Despite the high dose of *B. henselae* used in this experiment, bacteremia was only identified at PID 3 in one of the two high-dose ferrets, even with the use of enrichment blood culture. The ferrets’ culture and serological results in this study were very similar to previously published results from the experimental infection of a dog with culture-grown *B. henselae* SA2 [57]. The intradermal inoculation doses were chosen based on previous experiments with cats and dogs [26,33,76]. It appears that the high inoculum dose was sufficient to induce a robust serological response as well as possibly chronic disease and/or infection.

It has been suggested that *B. henselae* strain differences may influence the clinical manifestations of the disease [27,28,30,31,32,111]. For example, cats infected with a feline strain of *B. henselae* developed fever for nearly two weeks as well as relapsing bacteremia, whereas cats infected with *B. henselae* derived from a human patient showed no signs of clinical illness and no recurrence of bacteremia after initial infection, despite developing lower IgM and IgG titers [28]. The *B. henselae* strain chosen for inoculation in this study (SA2) is a feline strain initially isolated from a naturally infected cat; future studies could consider using human strains as well.

In addition to considering the impact of inoculum dose and strain, it is important to discuss the route of infection. *B. henselae* is typically transmitted to cats by *C. felis*, the cat flea, and to humans by inoculation of infected flea feces through the patient’s skin, often by scratches [1,3,4,112]. Because of the logistical and biosecurity challenges of an experimental model that relies on flea-borne transmission, most cat experimental models have used IV or ID inoculation of inoculum derived from cultured bacteria; in these studies, cats typically did not develop signs of clinical illness during acute or chronic infection, even when associated with persistent or relapsing bacteremia [25,26,33,36,113]. In this study, intradermal inoculation was used to mimic flea bites, but it is possible that bacterial virulence was diminished by using culture-grown organisms injected intradermally, rather than by transmission by the actual vector [114]. The importance of the vector in the transmission of *B. henselae* was illustrated in an experimental model in which cats infected with *B. henselae* by flea infestation developed signs of clinical illness, whereas cats infected by IV injection of the same *B. henselae* (CSU-1) strain grown in culture did not [30,32]. Similarly, cats experimentally infected by inoculation of *B. henselae*-infected flea feces developed clinical signs, long-duration bacteremia, and histopathological evidence of disease in multiple organ systems [27]. Indeed, ferrets are also susceptible to *C. felis* infestation [115], so it is possible to consider this infection route for future studies. Exposure to *B. henselae* from actual flea bites or inoculation of infected flea feces in future studies would more accurately mimic natural transmission and may change the disease outcomes compared to those reported in this study.

Considering the difficulties of maintaining a live flea colony to use for infection, some previous studies have also used blood transfusion from naturally infected cats to induce experimental infection [38,39,113]. In experimental models using cats infected by transfusion of blood from other infected cats, clinical signs were minimal but there was evidence of long-duration bacteremia, and histologic lesions in multiple organ systems [38]. Developing a ferret model that most closely mimics transmission in nature is of critical importance but establishing a reliable mode of transmission presents a number of challenges for future investigators.

There are several limitations of this study. The small number of animals included in this pilot study allowed for only two controls and four infected ferrets; future work on this potential model should increase the sample size to account for individual variation in health status. *B. henselae* DNA was only rarely detected in infected ferrets, and histopathological lesions were variable and not correlated with molecular detection, making it difficult to draw definitive conclusions on the pathophysiology of *Bartonella* infection even in this model animal. An unexpected positive qPCR result in one of the DNA extracts from the spleen of a control ferret suggests potential contamination, occult infection, or horizontal transmission, highlighting the critical importance of biosecurity and strict control measures in housing and handling when studying this pathogen. The use of intradermal inoculation may not accurately mimic natural transmission, and strain differences may influence clinical manifestations.

## 5. Conclusions

In conclusion, ferrets infected with *B. henselae* by intradermal developed circulating antibodies against *B. henselae* and had histologic evidence of inflammation in the liver, heart, and brain organs known to be potentially affected in human bartonellosis. Despite the limitations identified in this pilot study, ferrets could be further considered as an alternative to existing laboratory animal models of chronic *B. henselae* infection. Future studies should focus on pioneering a naturalistic model of infection using flea transmission, to determine whether or to what extent vector transmission differs from experimental intradermal needle inoculation and attempt to recapitulate the clinical manifestations, immune response, and a transmission pathway involved in human disease.

## Data Availability

The original contributions presented in the study are included in the article, further inquiries can be directed to the corresponding author.

## References

[1] Cheslock M.A., Embers M.E. (2019). Human Bartonellosis: An Underappreciated Public Health Problem?. Trop. Med. Infect. Dis..

[2] McCormick D.W., Rassoulian-Barrett S.L., Hoogestraat D.R., Salipante S.J., SenGupta D., Dietrich E.A., Cookson B.T., Marx G.E., Lieberman J.A. (2023). *Bartonella* spp. Infections Identified by Molecular Methods, United States. Emerg. Infect. Dis..

[3] Taber R., Pankowski A., Ludwig A.L., Jensen M., Magsamen V., Lashnits E. (2022). Bartonellosis in Dogs and Cats, an Update. Vet. Clin. N. Am. Small Anim. Pract..

[4] Regier Y., O’rourke F., Kempf V.A. (2016). *Bartonella* spp.—A chance to establish One Health concepts in veterinary and human medicine. Parasites Vectors.

[5] Qurollo B. (2019). Feline Vector-Borne Diseases in North America. Vet. Clin. N. Am. Small Anim. Pract..

[6] Pizzuti M., Bailey P., Derrick C., Albrecht B., Carr A.L., Covington E.W., Deri C.R., Green S.B., Hayes J., Hobbs A.L. (2024). Epidemiology and treatment of invasive Bar-tonella spp. infections in the United States. Infection.

[7] Okaro U., Addisu A., Casanas B., Anderson B. (2017). *Bartonella* Species, an Emerging Cause of Blood-Culture-Negative Endocarditis. Clin. Microbiol. Rev..

[8] Edouard S., Nabet C., Lepidi H., Fournier P.-E., Raoult D. (2015). *Bartonella*, a common cause of endocarditis: A report on 106 cases and review. J. Clin. Microbiol..

[9] Godfrey R., Curtis S., Schilling W.H., James P.R. (2020). Blood culture negative endocarditis in the modern era of 16S rRNA sequencing. Clin. Med..

[10] Allizond V., Costa C., Sidoti F., Scutera S., Bianco G., Sparti R., Banche G., Dalmasso P., Cuffini A.M., Cavallo R. (2019). Serological and molecular detection of *Bartonella henselae* in specimens from patients with suspected cat scratch disease in Italy: A comparative study. PLoS ONE.

[11] Nelson C.A., Moore A.R., Perea A.E., Mead P.S. (2018). Cat scratch disease: U.S. clinicians’ experience and knowledge. Zoonoses Public Health.

[12] Nelson C.A., Saha S., Mead P.S. (2016). Cat-Scratch Disease in the United States, 2005–2013. Emerg. Infect. Dis..

[13] Canneti B., Cabo-López I., Puy-Núñez A., García García J.C., Cores F.J., Trigo M., Suárez-Gil A.P., Rodriguez-Regal A. (2019). Neurological presentations of *Bartonella henselae* infection. Neurol. Sci..

[14] Shi J., Danesh-Meyer H.V., Bhatti M.T. (2025). Neuroretinitis: A comprehensive review on aetiologies, clinical manifestations, and treatment options. Eye.

[15] Jurja S., Stroe A.Z., Pundiche M.B., Docu Axelerad S., Mateescu G., Micu A.O., Popescu R., Oltean A., Docu Axelerad A. (2022). The Clinical Profile of Cat-Scratch Disease’s Neu-ro-Ophthalmological Effects. Brain Sci..

[16] Iralu J., Bai Y., Crook L., Tempest B., Simpson G., McKenzie T., Koster F. (2006). Rodent-associated *Bartonella* febrile illness, southwestern United States. Emerg. Infect. Dis..

[17] Ramos J.M., Perez-Tanoira R., Martin-Martin I., Prieto-Perez L., Tefasmariam A., Tiziano G., Escudero R., Gil-Zamorano J., Gil-Gil H., Gorgolas M. (2019). Arthropod-Borne Bacteria Cause Nonmalarial Fever in Rural Ethiopia: A Cross-Sectional Study in 394 Patients. Vector-Borne Zoonotic Dis..

[18] Kosoy M., Bai Y., Sheff K., Morway C., Baggett H., Maloney S.A., Boonmar S., Bhengsri S., Dowell S.F., Sitdhirasdr A. (2010). Identification of *Bartonella* infections in febrile human patients from Thailand and their potential animal reservoirs. Am. J. Trop. Med. Hyg..

[19] Hou S.L., Idris N., Tay S.T. (2022). Serological review of *Bartonella henselae* and *Bartonella* quintana infection among Malaysian pa-tients with unknown causes of febrile illnesses. Trop. Biomed..

[20] Bush J.C., Robveille C., Maggi R.G., Breitschwerdt E.B. (2024). Neurobartonelloses: Emerging from obscurity!. Parasites Vectors.

[21] Nawrocki C.C., Max R.J., Marzec N.S., Nelson C.A. (2020). Atypical manifestations of cat-scratch disease, United States, 2005–2014. Emerg. Infect. Dis..

[22] Beydon M., Rodriguez C., Karras A., Cez A., Rafat C., Jourde-Chiche N., Fain O., Philipponnet C., Puéchal X., Dossier A. (2022). *Bartonella and Coxiella* infections presenting as systemic vasculitis: Case series and review of literature. Rheumatology.

[23] Shamekhi Amiri F. (2017). Bartonellosis in Chronic Kidney Disease: An Unrecognized and Unsuspected Diagnosis. Ther. Apher. Dial..

[24] Domingos Grilo R., Madureira M., Reis Melo A., Tavares M. (2024). Cat-scratch disease: A rare cause of osteomyelitis. BMJ Case Rep..

[25] Guptill L., Slater L.N., Wu C.C., Lin T.L., Glickman L.T., Welch D.F., Tobolski J., HogenEsch H. (1998). Evidence of reproductive failure and lack of perinatal transmission of *Bartonella henselae* in experimentally infected cats. Vet. Immunol. Immunopathol..

[26] Guptill L., Slater L., Wu C., Lin T., Glickman L.T., Welch D.F., HogenEsch H. (1997). Experimental infection of young specific pathogen-free cats with *Bartonella henselae*. J. Infect. Dis..

[27] Foil L., Andress E., Freeland R.L., Roy A.F., Rutledge R., Triche P.C., O’Reilly K.L. (1998). Experimental Infection of Domestic Cats with Bar-tonella *henselae* by Inoculation of Ctenocephalides fells (Siphonaptera: Pulicidae) Feces. J. Med. Entomol..

[28] Yamamoto K., Chomel B.B., Kasten R.W., Hew C.M., Weber D.K., Lee W.I. (2002). Experimental infection of specific pathogen free (SPF) cats with two different strains of *Bartonella henselae* type I: A comparative study. Vet. Res..

[29] Chomel B.B., Kasten R.W., Stuckey M.J., Breitschwerdt E.B., Maggi R.G., Henn J.B., Koehler J.E., Chang C.-C. (2014). Experimental infection of cats with Afipia felis and various *Bartonella* species or subspecies. Vet. Microbiol..

[30] Lappin M.R., Davis W.L., Hawley J.R., Brewer M., Morris A., Stanneck D. (2013). A flea and tick collar containing 10% imidacloprid and 4.5% flumethrin prevents flea transmission of *Bartonella henselae* in cats. Parasites Vectors.

[31] Ficociello J., Bradbury C., Morris A., Lappin M. (2011). Detection of *Bartonella henselae* IgM in Serum of Experimentally Infected and Naturally Exposed Cats. J. Vet. Intern. Med..

[32] Bradbury C.A., Lappin M.R. (2010). Evaluation of topical application of 10% imidacloprid–1% moxidectin to prevent *Bartonella henselae* transmission from cat fleas. J. Am. Vet. Med. Assoc..

[33] Abbott R.C., Chomel B.B., Kasten R.W., Floyd-Hawkins K.A., Kikuchi Y., Koehler J.E., Pedersen N.C. (1997). Experimental and natural infection with *Bartonella henselae* in domestic cats. Comp. Immunol. Microbiol. Infect. Dis..

[34] Chomel B.B., Kasten R.W., Floyd-Hawkins K., Chi B., Yamamoto K., Roberts-Wilson J., Gurfield A.N., Abbott R.C., Pedersen N.C., Koehler J.E. (1996). Experimental Transmission of Bar-tonella *henselae* by the Cat Flea. J. Clin. Microbiol..

[35] Zanutto M.D.S., Mamizuka E.M., Raiz-Júnior R., Lima T.M., Diogo C.L., Okay T.S., Hagiwara M.K. (2001). Experimental infection and horizontal transmission of *Bartonella henselae* in domestic cats. Rev. Inst. Med. Trop. São Paulo.

[36] Guptill L., Slater L., Wu C.-C., Glickman L.T., Lin T.-L., Welch D.F., Crippen J.T., HogenEsch H. (1999). Immune response of neonatal specific pathogen-free cats to experimental infection with *Bartonella henselae*. Vet. Immunol. Immunopathol..

[37] Greene C.E., Mcdermott M., Jameson P.H., Atkins C.L., Marks A.M. (1996). *Bartonella henselae* infection in cats: Evaluation during pri-mary infection, treatment, and rechallenge infection. J. Clin. Microbiol..

[38] Kordick D.L., Brown T.T., Shin K., Breitschwerdt E.B. (1999). Clinical and pathologic evaluation of chronic *Bartonella henselae* or Bar-tonella clarridgeiae infection in cats. J. Clin. Microbiol..

[39] Kordick D.L., Breitschwerdt E.B. (1997). Relapsing bacteremia after blood transmission of *Bartonella henselae* to cats. Am. J. Vet. Res..

[40] Koesling J., Aebischer T., Falch C., Schulein R., Dehio C. (2001). Cutting Edge: Antibody-Mediated Cessation of Hemotropic Infection by the Intraerythrocytic Mouse Pathogen *Bartonella grahamii*. J. Immunol..

[41] Deng H.K., Le Rhun D., Lecuelle B., Le Naour E., Vayssier-Taussat M. (2012). Role of the spleen in *Bartonella* spp. infection. FEMS Immunol. Med. Microbiol..

[42] Siewert L.K., Fromm K., Dehio C., Pinschewer D.D. (2024). Cutting Edge: Redundant Roles for MHC Class II-, CD1d-, and MR1-restricted T Cells in Clearing *Bartonella* Infection. J. Immunol..

[43] Fromm K., Boegli A., Ortelli M., Wagner A., Bohn E., Malmsheimer S., Wagner S., Dehio C. (2022). *Bartonella* taylorii: A Model Organism for Studying *Bartonella* Infection in vitro and in vivo. Front. Microbiol..

[44] de Almeida A.R., Vieira-Damiani G., da Silva M.N., Lania B.G., Soares T.C.B., Drummond M.R., Lins K.d.A., Ericson M.E., Gupta K., Velho P.E.N. (2019). *Bartonella henselae* Infection in Sickle Cell Disease Mice Is Associated with Hyperalgesia. Vector-Borne Zoonotic Dis..

[45] da Silva M.N., Vieira-Damiani G., Ericson M.E., Gupta K., de Almeida A.R., Drummond M.R., Soares T.C.B., Lania B.G., Gilioli R., Velho P.E.N.F. (2017). Acute and Late *Bartonella henselae* Murine Model Infection. Vector-Borne Zoonotic Dis..

[46] Silva M.N., Vieira-Damiani G., Ericson M.E., Gupta K., Gilioli R., de Almeida A.R., Drummond M.R., Lania B.G., Lins K.d.A., Soares T.C.B. (2016). *Bartonella henselae* transmission by blood transfusion in mice. Transfusion.

[47] Karem K.L., Dubois K.A., McGill S.L., Regnery R.L. (1999). Characterization of *Bartonella henselae*-specific immunity in BALB/c mice. Immunology.

[48] Siewert L.K., Dehio C., Pinschewer D.D. (2022). Adaptive immune defense prevents *Bartonella* persistence upon trans-placental transmission. PLoS Pathog..

[49] Lima A., Cha B.J., Amin J., Smith L.K., Anderson B. (2014). Zebrafish Embryo Model of *Bartonella henselae* Infection. Zebrafish.

[50] Drut A., Bublot I., Breitschwerdt E.B., Chabanne L., Vayssier-Taussat M., Cadoré J.-L. (2014). Comparative microbiological features of *Bartonella henselae* infection in a dog with fever of unknown origin and granulomatous lymphadenitis. Med. Microbiol. Immunol..

[51] Morales S.C., Breitschwerdt E.B., Washabau R.J., Matise I., Maggi R.G., Duncan A.W. (2007). Detection of *Bartonella henselae* DNA in two dogs with pyogranulomatous lymphadenitis. J. Am. Vet. Med. Assoc..

[52] Gillespie T.N., Washabau R.J., Goldschmidt M.H., Cullen J.M., Rogala A.R., Breitschwerdt E.B. (2003). Detection of *Bartonella henselae* and *Bartonella* clarridgeiae DNA in hepatic specimens from two dogs with hepatic disease. J. Am. Vet. Med. Assoc..

[53] Ohad D.G., Morick D., Avidor B., Harrus S. (2010). Molecular detection of *Bartonella henselae* and *Bartonella* koehlerae from aortic valves of Boxer dogs with infective endocarditis. Vet. Microbiol..

[54] Tucker M.D., Sellon R.K., Tucker R.L., Wills T.B., Simonsen A., Maggi R.G., Breitschwerdt E.B. (2014). Bilateral mandibular pyogranulomatous lymphadenitis and pulmonary nodules in a dog with *Bartonella henselae* bacteremia. Can. Vet. J..

[55] Mexas A.M., Hancock S.I., Breitschwerdt E.B. (2002). *Bartonella henselae* and *Bartonella elizabethae* as Potential Canine Pathogens. J. Clin. Microbiol..

[56] Chomel B.B., Ermel R.W., Kasten R.W., Henn J.B., Fleischman D.A., Chang C.-C. (2014). Experimental infection of dogs with various *Bartonella* species or subspecies isolated from their natural reservoir. Vet. Microbiol..

[57] Balakrishnan N., Cherry N.A., Linder K.E., Pierce E., Sontakke N., Hegarty B.C., Bradley J.M., Maggi R.G., Breitschwerdt E.B. (2013). Experimental infection of dogs with Bartonella *henselae* and *Bartonella* vinsonii subsp. berkhoffii. Vet. Immunol. Immunopathol..

[58] Kim Y.-I., Kim S.-G., Kim S.-M., Kim E.-H., Park S.-J., Yu K.-M., Chang J.-H., Kim E.J., Lee S., Casel M.A.B. (2020). Infection and Rapid Transmission of SARS-CoV-2 in Ferrets. Cell Host Microbe.

[59] Belser J.A., Eckert A.M., Huynh T., Gary J.M., Ritter J.M., Tumpey T.M., Maines T.R. (2020). A Guide for the Use of the Ferret Model for Influenza Virus Infection. Am. J. Pathol..

[60] Wong J., Layton D., Wheatley A.K., Kent S.J. (2019). Improving immunological insights into the ferret model of human viral infec-tious disease. Influenza Other Respir. Viruses.

[61] Sigurdson C., Mathiason C., Perrott M., Eliason G., Spraker T., Glatzel M., Manco G., Bartz J., Miller M., Hoover E. (2008). Experimental Chronic Wasting Disease (CWD) in the Ferret. J. Comp. Pathol..

[62] Ludlow M., Rennick L.J., Nambulli S., de Swart R.L., Duprex W.P. (2014). Using the ferret model to study morbillivirus entry, spread, transmission and cross-species infection. Curr. Opin. Virol..

[63] da Fontoura Budaszewski R., von Messling V. (2016). Morbillivirus experimental animal models: Measles virus pathogenesis in-sights from canine distemper virus. Viruses.

[64] Pillet S., Svitek N., von Messling V. (2009). Ferrets as a model for morbillivirus pathogenesis, complications, and vaccines. Curr. Top. Microbiol. Immunol..

[65] McCallan L., Corbett D., Andersen P.L., Aagaard C., McMurray D., Thompson S., Strain S., McNair J. (2011). A New Experimental Infection Model in Fer-rets Based on Aerosolised Mycobacterium bovis. Vet. Med. Int..

[66] de Vries R.D., Ludlow M., de Jong A., Rennick L.J., Verburgh R.J., van Amerongen G., van Riel D., van Run P.R.W.A., Herfst S., Kuiken T. (2017). Delineating morbillivirus entry, dis-semination and airborne transmission by studying in vivo competition of multicolor canine distemper viruses in ferrets. PLoS Pathog..

[67] Pramod R.K., Atul P.K., Pandey M., Anbazhagan S., Mhaske S.T., Barathidasan R. (2024). Care, management, and use of ferrets in bio-medical research. Lab. Anim. Res..

[68] Carrasco S.E., Chomel B.B., Gill V.A., Doroff A.M., Miller M.A., Burek-Huntington K.A., Kasten R.W., Byrne B.A., Goldstein T., Mazet J.A. (2014). *Bartonella* spp. exposure in northern and southern sea otters in Alaska and California. Vector-Borne Zoonotic Dis..

[69] Carrasco S.E., Chomel B.B., Gill V.A., Kasten R.W., Maggi R.G., Breitschwerdt E.B., Byrne B.A., Burek-Huntington K.A., Miller M.A., Goldstein T. (2014). Novel *Bartonella* infection in northern and southern sea otters (Enhydra lutris kenyoni and Enhydra lutris nereis). Vet. Microbiol..

[70] Chinnadurai S.K., Birkenheuer A.J., Blanton H.L., Maggi R.G., Belfiore N., Marr H.S., Breitschwerdt E.B., Stoskopf M.K. (2010). Prevalence of selected vector-borne organisms and identification of *Bartonella* species DNA in North American river otters (Lontra canadensis). J. Wildl. Dis..

[71] Millán J., Proboste T., de Mera I.G.F., Chirife A.D., de la Fuente J., Altet L. (2016). Molecular detection of vector-borne pathogens in wild and domestic carnivores and their ticks at the human–wildlife interface. Ticks Tick-Borne Dis..

[72] Sepúlveda-García P., Raffo E., Medina-Vogel G., Muñoz F., Muñoz P., Alabí A., Navarrete-Talloni M.J., Gonçalves L.R., de Mello V.V.C., Machado R.Z. (2021). Molecular survey of *Bartonella* spp. and haemoplasmas in American minks (Neovison vison). Transbound. Emerg. Dis..

[73] Quinn J.H., Girard Y.A., Gilardi K., Hernandez Y., Poppenga R., Chomel B.B., Foley J.E., Johnson C.K. (2012). Pathogen and rodenticide exposure in Amer-ican badgers (Taxidea taxus) in California. J. Wildl. Dis..

[74] Gerrikagoitia X., Gil H., García-Esteban C., Anda P., Juste R.A., Barral M. (2012). Presence of *Bartonella* Species in Wild Carnivores of Northern Spain. Appl. Environ. Microbiol..

[75] Orellana-Rios J., Verdaguer-Diaz J.I., Opazo G., Leong B.C., Zett C., Smith R.T., Freund K.B. (2020). Not cat-scratch disease: *Bartonella henselae* neuroretinitis associated with non-feline pet mammals. IDCases.

[76] Yamamoto K., Chomel B., Kasten R., Chang C., Tseggai T., Decker P., Mackowiak M., Floyd-Hawkins K., Pedersen N. (1998). Homologous protection but lack of heterologous-protection by various species and types of *Bartonella* in specific pathogen-free cats. Vet. Immunol. Immunopathol..

[77] Neupane P., Sevala S., Balakrishnan N., Marr H., Wilson J., Maggi R., Birkenheuer A., Lappin M., Chomel B., Breitschwerdt E.B. (2020). Validation of *Bartonella henselae* Western Immunob-lotting for Serodiagnosis of Bartonelloses in Dogs. J. Clin. Microbiol..

[78] Species360 Zoological Information Management System ZIMS Expected Test Results for Mustela Putorius Furo. http://zims.species360.org.

[79] Oteo J.A., Maggi R., Portillo A., Bradley J., García-Álvarez L., San-Martín M., Roura X., Breitschwerdt E. (2017). Prevalence of *Bartonella* spp. by culture, PCR and serology, in veterinary personnel from Spain. Parasites Vectors.

[80] Portillo A., Maggi R., Oteo J.A., Bradley J., García-Álvarez L., San-Martín M., Roura X., Breitschwerdt E. (2020). *Bartonella* spp. Prevalence (Serology, Culture, and PCR) in Sanitary Workers in La Rioja Spain. Pathogens.

[81] Varanat M., Maggi R.G., Linder K.E., Horton S., Breitschwerdt E.B. (2009). Cross-contamination in the Molecular Detection of *Bartonella* from Paraffin-embedded Tissues. Vet. Pathol..

[82] Duncan A.W., Maggi R.G., Breitschwerdt E.B. (2007). A combined approach for the enhanced detection and isolation of *Bartonella* species in dog blood samples: Pre-enrichment liquid culture followed by PCR and subculture onto agar plates. J. Microbiol. Methods.

[83] Maggi R.G., Birkenheuer A.J., Hegarty B.C., Bradley J.M., Levy M.G., Breitschwerdt E.B. (2014). Comparison of serological and molecular panels for diagnosis of vector-borne diseases in dogs. Parasites Vectors.

[84] Okaro U., George S., Anderson B. (2021). What is in a cat scratch? Growth of *Bartonella henselae* in a biofilm. Microorganisms.

[85] Harms A., Dehio C. (2012). Intruders below the radar: Molecular pathogenesis of *Bartonella* spp.. Clin. Microbiol. Rev..

[86] Lehmer E.M., Lavengood K., Miller M., Rodgers J., Fenster S.D. (2018). Evaluating the Impacts of Coinfection on Immune System Function of the Deer Mouse (Peromyscus Maniculatus) Using Sin Nombre Virus and *Bartonella* as Model Pathogen Systems. J. Wildl. Dis..

[87] Kanık-Yüksek S., Gülhan B., Hürmüzlü S., Özkaya-Parlakay A., Güneş A., Oğuz-Erdoğan A.S., Tezer H. (2022). Challenges of the treatment of pediatric hepatosplenic bartonellosis: Case report and literature review. J. Infect. Dev. Ctries..

[88] Massei F., Gori L., Macchia P., Maggiore G. (2005). The Expanded Spectrum of Bartonellosis in Children. Infect. Dis. Clin. N. Am..

[89] Arisoy E.S., Correa A.G., Wagner M.L., Kaplan S.L. (1999). Hepatosplenic cat-scratch disease in children: Selected clinical features and treatment. Clin. Infect. Dis..

[90] VanderHeyden T.R., Yong S.L., Breitschwerdt E.B., Maggi R.G., Mihalik A.R., Parada J.P., Fimmel C.J. (2012). Granulomatous hepatitis due to *Bartonella henselae* infection in an immunocompetent patient. BMC Infect. Dis..

[91] Laham F.R., Kaplan S.L. (2008). Hepatosplenic cat-scratch fever. Lancet Infect Dis..

[92] Stroescu R.F., Chisavu F., Steflea R.M., Doros G., Bizerea-Moga T.-O., Vulcanescu D.D., Marti T.D., Boru C., Avram C.R., Gafencu M. (2024). A Retrospective Analysis of Systemic *Bartonella henselae* Infection in Children. Microorganisms.

[93] Liston T.E., Koehler J.E. (1996). Granulomatous hepatitis and necrotizing splenitis due to *Bartonella henselae* in a patient with cancer: Case report and review of hepatosplenic manifestations of *Bartonella* infection. Clin. Infect. Dis..

[94] Khonde P., Byrnes K. (2023). The Brief Case: Granulomatous hepatitis in an immunocompromised patient. J. Clin. Microbiol..

[95] Lamps L.W., Scott M.A. (2004). Cat-scratch disease: Historic, clinical, and pathologic perspectives. Am. J. Clin. Pathol..

[96] García J.C., Núñez M.J., Castro B., Fernández J.M., Portillo A., Oteo J.A. (2014). Hepatosplenic cat scratch disease in immunocompetent adults: Report of 3 cases and review of the literature. Medicine.

[97] Agrawal S.K., Das P., Shalimar, Swatantra G., Chaudhry R. (2019). Multifocal hepatic abscesses in immunocompetent patient due to *Bartonella henselae*: Case report with review of literature. Indian J. Med. Microbiol..

[98] Varanat M., Broadhurst J., Linder K.E., Maggi R.G., Breitschwerdt E.B. (2012). Identification of *Bartonella henselae* in 2 Cats With Pyogranulomatous Myocarditis and Diaphragmatic Myositis. Vet. Pathol..

[99] Elsmo E.J., Fenton H., Cleveland C.A., Shock B., Cunningham M., Howerth E.W., Yabsley M.J. (2018). Necrotizing interstitial pneumonia and suppurative myocarditis associated with *Bartonella henselae* infection in three Florida pumas. J. Vet. Diagn. Investig..

[100] Donovan T., Fox P., Balakrishnan N., Ericson M., Hooker V., Breitschwerdt E. (2017). Pyogranulomatous Pancarditis with Intramyocardial *Bartonella henselae* San Antonio 2 (*Bh*SA2) in a Dog. J. Vet. Intern. Med..

[101] Gerber J., Johnson J., Scott M., Madhusudhan K. (2002). Fatal Meningitis and Encephalitis Due to *Bartonella henselae* Bacteria. J. Forensic Sci..

[102] Fouch B., Coventry S. (2007). A case of fatal disseminated *Bartonella henselae* infection (cat-scratch disease) with encephalitis. Arch. Pathol. Lab. Med..

[103] Pinto V.L., Curi A.L., Pinto A.D., Nunes E.P., Teixeira M.D., Rozental T., Favacho A.R., Lemos E.R., Bóia M.N. (2008). Cat scratch disease complicated with aseptic meningitis and neuroretinitis. Braz. J. Infect. Dis..

[104] Kassab I., Isada C., Azar M.M., Sarsam N., Jiang M., Camelo-Piragua S., Kaul D., Malinis M. (2022). Into the unknown: Diagnosing mysterious brain lesions. Transpl. Infect. Dis..

[105] Lins K.D., Drummond M.R., Velho P.E. (2019). Cutaneous manifestations of bartonellosis. An. Bras. Dermatol..

[106] Lashnits E., Neupane P., Maggi R.G., Linder K.E., Bradley J.M., Balakrishnan N., Southern B.L., McKeon G.P., Chandrashekar R., Breitschwerdt E.B. (2020). Detection of *Bartonella* spp. in dogs after infection with Rickettsia rickettsii. J. Vet. Intern. Med..

[107] Santos L.S., Martins S.A., Scheffer F.R., Maekawa A.S., Silva R.D., Araújo G.R., Velho P.E., Drummond M.R. (2024). Investigation of natural infection of BALB C mice by *Bartonella henselae*. Braz. J. Infect Dis..

[108] Dong Y., Alhaskawi A., Zou X., Zhou H., Ezzi S.H.A., Kota V.G., Abdulla M.H.A.H., Olga A., Abdalbary S.A., Lu H. (2024). Post-COVID reactivation of latent *Bartonella henselae* infection: A case report and literature review. BMC Infect. Dis..

[109] Aubry A., Corvilain E., Ghelfenstein-Ferreira T., Camelena F., Meignin V., Berçot B., Le Goff J., Salmona M. (2024). Unmasking *Bartonella henselae* infection in the shadows of long COVID thanks to clinical metagenomics. Eur. J. Clin. Microbiol. Infect. Dis..

[110] Bush J.C., Maggi R.G., Breitschwerdt E.B. (2023). Viability and Desiccation Resistance of *Bartonella henselae* in Biological and Non-Biological Fluids: Evidence for Pathogen Environmental Stability. Pathogens.

[111] Yamamoto K., Chomel B.B., Kasten R.W., Hew C.M., Weber D.K., Lee W.I., Koehler J.E., Pedersen N.C. (2003). Infection and re-infection of domestic cats with various *Bartonella* species or types: *B. henselae* type I is protective against heterologous challenge with *B. henselae* type II. Vet. Microbiol..

[112] Rust M.K. (2017). The Biology and Ecology of Cat Fleas and Advancements in Their Pest Management: A Review. Insects.

[113] Lappin M.R., Fitzgerald R. (2024). Pradofloxacin for Treatment of *Bartonella henselae* in Experimentally Inoculated Cats. Pathogens.

[114] Jarrett C.O., Sebbane F., Adamovicz J.J., Andrews G.P., Hinnebusch B.J. (2004). Flea-Borne Transmission Model To Evaluate Vaccine Efficacy against Naturally Acquired Bubonic Plague. Infect. Immun..

[115] Hutchinson M.J., Jacobs D.E., Mencke N. (2001). Establishment of the cat flea (*Ctenocephalides felis felis*) on the ferret (*Mustela putorius furo*) and its control with imidacloprid. Med. Vet. Èntomol..

